# The Potential Effect of β-Ionone and β-Damascenone on Sensory Perception of Pinot Noir Wine Aroma

**DOI:** 10.3390/molecules26051288

**Published:** 2021-02-27

**Authors:** Elizabeth Tomasino, Shiloh Bolman

**Affiliations:** Department of Food Science and Technology, Oregon State University, 100 Wiegand Hall, Corvallis, OR 97333, USA; bolmans@onid.oregonstate.edu

**Keywords:** norisoprenoid, aroma, triangle test, oregon wine, New Zealand wine, descriptive analysis

## Abstract

Volatile compounds are responsible for driving the aroma of wine. Because of their low perception thresholds, norisoprenoids may play an important role in wine aroma. Studies have shown that β-damascenone may act as an aroma enhancing compound. However, the direct impact on wine aroma is unclear. Our study examined the direct impact of β-ionone and β-damascenone on the aroma sensory perception of Pinot noir wines. Triangle tests were used to determine if assessors could distinguish between wines with varying concentrations of β-ionone and β-damascenone in three different Pinot noir wine matrixes. Descriptive analysis was performed on these treatments, perceived as different in triangle tests. Results show that β-ionone acts as a significant contributor to aromas in Pinot noir wine, as individuals could differentiate both the low and high concentration wines from the control. How β-ionone impacted wine aroma depends on the wine matrix, as different aroma descriptors were affected based on the model wine used, resulting in floral, red berry or dark berry aromas. The effect of β-damascenone on Pinot noir aroma was less clear, as perception seems to be heavily influenced by wine matrix composition. This study contributes to our understanding of the complex chemical causation of fruity aromas in Pinot noir wine.

## 1. Introduction

β-ionone and β-damascenone are C-13 norisoprenoid volatile aroma compounds that are found in grapes and their respective wines. There is considerable interest in the effect of these compounds on wine aroma as, anecdotally, it is thought that higher levels of these compounds may improve wine quality. Specifically, there is interest in increasing the content in grapes and wine above what is normally found through using vineyard management practices. These compounds are varietal aroma compounds found in grapes and derived from the breakdown of carotenoids through enzymatic and acid catalyzed hydrolysis [1]. This breakdown results in the formation of free form volatile aroma compounds or non-volatile glycoconjugates within the vacuoles of grape skin cells [2]. β-ionone is a secondary metabolite derived from β-carotene [3]. β-damascenone is a breakdown product of different aglycons [4] and neoxanthin [5].

β-ionone is known for contributing a violet aroma to red wine [1,3,6]. The sensory effect of β-damascenone is less clear. Descriptors of baked/cooked apples, honey, and flowery fruity aromas have been reported [1,7]. Both of these compounds are thought to have a significant effect on the perception of a wine’s aroma because of their low odor thresholds. The threshold for β-ionone was found to be 0.007 μg/L in water [1] and 0.09 μg/L in a model wine solution [3]. The threshold for β-damascenone was found to be 0.002 μg/L in water and 0.05 μg/L in a hydroalcoholic solution with ethanol in the range of 10–12% [8]. However, it should be noted that thresholds determined in a simple matrix are not representative of the thresholds in more complex matrixes, such as wine [9,10].

Several studies have examined the effect of the non-volatile portion of the wine matrix on the perception of aroma. It is known that some portions of the non-volatile matrix, such as the ethanol concentration and catechin content, have an impact on the aroma of red wines [11,12,13]. However, the effect of the non-volatile matrix has not been studied extensively in Pinot noir. Additionally, a fact not addressed in previous work on norisoprenoid perception in wine, is that there is a specific anosmia associated with β-ionone perception [14,15]. This anosmia could significantly impact the perception of Pinot noir wine aroma quality. This sparks the question: does the impact of β-ionone and β-damascenone in the red wines studied previously hold true for Pinot noir? The purpose of this study is to examine whether or not the direct effect of β-ionone and β-damascenone on a Pinot noir wine aroma could be considered ubiquitous to all Pinot noirs, or if the direct effect is dependent upon a wine matrix effect based on the composition of the individual wine matrix.

## 2. Results

### 2.1. Triangle Tests

#### Study 1

Of the 18 total concentrations of β-ionone and β-damascenone tested across the three wine matrixes, three where found to be not significant (Table 1 and Table 2). All concentrations of β-ionone were found to be statistically significant across all three model wines. Although the *p*-value level did differ depending on the wine matrix. Unlike β-ionone, perception of β-damascenone was altered based on the wine matrix used. As can be seen in Table 3, panelists could perceive β-damascenone at all tested concentration levels for MW1 but not for MW2 or MW3. For MW2, β-damascenone was only perceived at 3× levels (21 μg/L) and for MW3, β-damascenone was perceived at high (6 μg/L) and 3× (21 μg/L) concentrations.

### 2.2. Descriptive Analysis

Descriptive analysis result showed that wine aroma quality changed based on the compound and concentration in wine and if it was altered by the wine matrix. Table 4 shows the ANOVA results for each of the terms used in the analysis. The aroma of dark berries was found to be altered by the compound and concentration, while additionally, an interaction was also found with the wine used. Apparently, there was a difference in vanilla between the model wines used.

**Figure 1:**
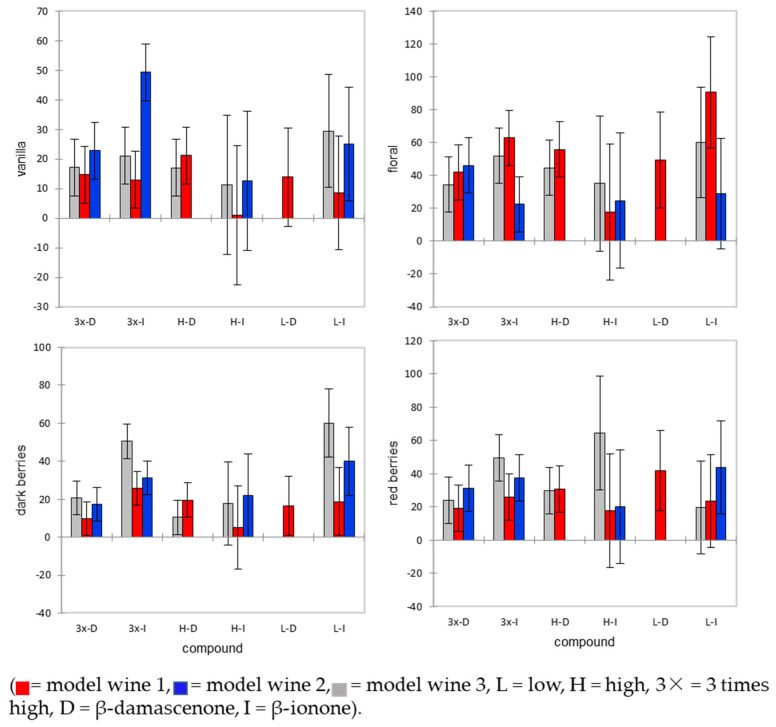
Means and standard deviation for each sensory attribute by compound concentration and model wine.

It is also possible to see how the addition of β-ionone and β-damscenone alters aroma by the means and standard deviations for each attribute evaluated (Figure 1). The main difference in vanilla aroma between the model wines was that MW1 had a higher intensity of vanilla. While not significant at α = 0.05, 3×-I in MW2 showed an increased intensity of vanilla aroma. For dark berry aroma, L-I and 3×-I were different from the other compound additions. Additionally, within these compound groups, the perception of dark berries was different based on the model wine. For example, within L-I, MW1 had a significantly lower intensity of dark berries than MW2 and MW3.

### 2.3. Chemical Analysis

While it was not possible to run statistical analyses (ANOVA) on the chemical results—as only singular measurements per wine were made—differences between the wines for all chemicals measured, except for molecular SO_2_, where determined.

## 3. Discussion

The concentrations of β-ionone and β-damascenone used were the lowest (L) and highest (H) recorded concentrations found in Pinot noir [16,17,18]. The objective was to examine the direct effect of each compound at concentrations found in Pinot noir wines. It is anecdotally thought that higher concentrations of β-ionone and β-damascenone can elicit a positive effect on the aroma characteristics of red wine. The highest concentration in this study was three times (3×) higher than that currently reported in previous work (Table 1). This concentration is highly exaggerated and was used to determine the direct effect of β-ionone and β-damascenone at a concentration higher than would be found in grapes, as management techniques have been used to attempt to increase the amount of these compounds in grapes and wine [19]. The use of the model wines, as described in the Materials and Methods Section, allowed for controls that contained non-detectable amounts of the both β-ionone and β-damascenone. Additionally, the model wines were used as they maintained the non-volatile composition of the wines and still contained other wine aroma compounds found in Pinot noir. This has been proven as an effective strategy in other sensory studies, as reconstitution studies have been found to be problematic in other pieces of work [6,20]. Other studies found that the non-volatile portion of the matrix has a profound effect on the perception of fruity and red berry aromas in red wine [11,13].

Results showed that β-damascenone and β-ionone altered the aroma perception of Pinot noir wines at the three chosen concentrations. In particular, the low concentration of β-ionone altered the aroma of all three Pinot noir model wines. The low concentration (0.1 µg/L) is the current known threshold of 0.09 µg/L in water [3]. However, compound thresholds in mixtures such as wine tend to be higher than in simple solutions such as water [10]. Although these wines with added β-ionone could be differentiated from the controls in triangle tests, the results from the descriptive analysis show that the wine matrix also plays a role in perception. For example, L-I in MW1 increased the floral aroma, but in MW2, red berries had the greatest intensity for L-I, and in MW3, the dark berry aroma was most prevalent. β-ionone has been known to positively enhance the red berry aromas in Spanish red wines [11] and the descriptive aroma of the pure compound of β-ionone is a floral aroma [21]. β-ionone was found to alter red berry aromas in combination with esters [11]. The exaggerated concentration of β-ionone tested (3×) elicited a negative response in perception from the winemakers. The professional winemakers used negative descriptors, such as “rotten potpourri” and “musty” to describe samples that contained this exaggerated concentration (data not shown). This type of change in the type of aroma perceived was found in most of the compound concentrations and model wines, suggesting that some type of interaction with other aroma compounds was also altering the aroma of the wines.

There has been much discussion in the literature about the impact of β-damascenone on wine aroma. Some studies suggest that β-damascenone plays a large role in the red wine aroma [22], while others suggest that it is an aroma enhancer compound that elevates the effect of other aroma constituents [7]. The differences in the wine matrix studied could explain the inconsistent findings in the literature about the presence of β-damascenone in wine. Unlike the β-ionone results, the β-damascenone results showed a greater influence of the wine matrix, as the different added concentrations could not be told apart from the controls across the different model wines (Table 3). The differences in aroma descriptors based on the β-damascenone concentrations were not found to be statistically significant (Figure 1). β-damascenone appeared to have the highest intensity for floral aromas, followed by red berry aromas. Our results suggest that the wine matrix does influence the perception of β-damascenone.

The effect of the non-volatile impact of the wine matrix on the aroma of wines is still unclear. Several studies have examined the effects of ethanol to depress the perceptions of fruity aromas in red wines [10,12]. The three wines used in this study all had the same ethanol content of 14%. Robinson et al. [12] also found that several non-volatile wine matrix compounds, including catechin, play a significant role in the perception of β-ionone and β-damascenone in red wines. The results of the tannin analysis indicate that there is a significant difference in catechin content across the three model wines. MW1 contained nearly twice as much catechin as MW2. The consumers response to these wines indicated that MW1 was the most aromatically active, as both β-ionone and β-damascenone had a significant effect on the aroma perception at all concentrations (Table 2 and Table 3). MW2 had the least amount of catechin and was the least aromatically active wine, with no significant values for L and H concentrations of β-damascenone. These results indicate that, while levels of catechin in wine do not seem to influence the perception of β-ionone, they may be highly influential in the perception of β-damascenone. Further studies are needed to determine if the perception differences of β-damascenone are caused by the volatile matrix of the wine or the non-volatile matrix.

Again, one of the main reasons for testing the 3× concentrations of both compounds was to determine if increasing the content had a positive or negative impact on sensory element of the wine, as management techniques have been used to increase the content in grapes and wine. Viticultural practices that alter the vine microclimate have shown to have an effect on the norisoprenoid concentrations of grapes and wines [23,24]. However, the results presented in the literature remain inconsistent. Feng et al. [24] showed that there was strong evidence to indicate that there was a connection between increased sun exposure to clusters in raising the level of β-damascenone in Pinot noir. However, Lee et al. [23] showed that norisoprenoid levels were strongly influenced by leaf layer number and were independent of sun exposure in Cabernet Sauvignon. Another study by Song et al. [25] reported that sun exposure had little positive effect on β-ionone and no effect on β-damascenone. In this previous study, it was discussed that inconsistent timings of treatments that reduced leaf layers and increased sun exposure to the cluster could account for this discrepancy. It is also possible that β-damascenone level increased because there were more carotenoid precursors available in the unripe grapes to later be broken down [24]. A more recent study showed that sunlight was found to include the accumulation of β-ionone but not β-damascenone [26].

While it appears to be possible to alter the content of β-ionone and β-damascenone in grapes and wine, our sensory results suggest that to achieve specific sensory qualities, it is important to focus on practices that alter other wine compositional factors. Additionally, our results also show that in order to predict sensory qualities in Pinot noir, it is necessary to figure out the matrix interactions that are altering the sensory qualities of these two compounds. Alone, these compounds are not predictors of specific aroma qualities.

We would like to note that the influence of the panelists sensitivity to compounds is important. After this study was completed, we went back to attempt to recruit wine consumers that could not perceive β-ionone at low concentrations. It would be interesting to see how this altered the perception of these compounds in wine. We were not able to recruit enough wine consumers that were insensitive to β-ionone to run the sensory trials at the desired power and alpha-level. This raises an interesting hypothesis for future exploration into the sensitivity of wine consumers to aromas.

## 4. Materials and Methods

### 4.1. Samples and Reagents

β-ionone (96%,) and β-damascenone (90%) were supplied by Sigma-Aldrich (ST. Louis, MO, USA). Ethanol was obtained from Pharmco-Aaper (Vancouver, WA, USA). Milli-Q water was obtained from a Millipore Continental water system (EMD-Millipore, Billerica, MA, USA).

### 4.2. Aroma Standards

Stock standards of each compound were prepared in 14% (*v*/*v*) aqueous ethanol (see Table 1). Stock standards were stored at −18 °C until used. They were defrosted, inverted 3 times to mix, and then added to wines to achieve specific concentrations (Table 1 and Table 2). The standards were added to the model wines 1 h before pouring and the wines were poured no more than 15 min before the start of each sensory session, using a new bottle of wine per session. The highest and lowest concentrations of β-ionone and β-damascenone used in this study were based off of concentrations found in literature [8,16,18,25]. An additional concentration was used at three times the highest concentration found in the literature, in order to determine if higher concentrations of these aroma compounds directly impact the perception of the wine in a positive or negative manner (Table 1 and Table 2).

### 4.3. Model Wine Formation

Three commercial Pinot Noirs were used in this study: two from Oregon (MW1 and MW2) and one from New Zealand (MW3). The wines were selected due to their low levels of β-ionone and β-damascenone, determined by GCMS (data not shown). Model wines were created to obtain wine solutions with undetectable levels of these two compounds, using a rotary evaporator (Büchi Rotavapor R-205, 35 °C and 180 rpm, Newcastle, DE, USA). A total of 100 mL of the model wines were processed as samples in the rotary evaporator, 35 °C and 180 rp, using a vacuum pump (Chemglass CG-4812-30, 17 LPM, 10 Torr) for about 25 min; the time required to remove all aromatics from the wines. This duration of time and loss of primary aromas was determined by trained professionals in a preliminary sensory panel and GCMS analysis (data not shown). All rotovaped samples were reconstituted back to their original volume at 14% alcohol content using ethanol and deionized water, to ensure a similar non-volatile matrix as the starting wines. The model wines contained 80% normal wine and 20% of the dearomatized wine. Information and basic wine parameters for all three model wines can be found in Table 4.

### 4.4. Screening for β-Ionone Sensitivity

There is a specific anosmia associated with β-ionone detection [15,27] which has the potential to influence sensory results in this study. Therefore, individuals were screened for their β-ionone sensitivity prior to the formal sensory sessions. β-ionone at low (100 µg/L) and high (0.5 mg/L) concentrations in deionized water were used in triangle tests for screening [28]. The trials consisted of two triangle tests in clear ISO glasses (ISO, 1977). The control samples consisted of 20 mL of deionized water. Set one contained the low concentration of β-ionone and set two contained the high concentration (0.5 mg/L). Only panelists who could accurately detect both the high and low concentrations of the β-ionone were invited to participate in the full study.

### 4.5. Triangle Tests

ISO triangle test methodology was followed [28]. In total, 26 females and 16 males (21–65 years old) participated. Inclusion criteria were as follows: participants had to consume red wine on average at least once a week; be over 21 years of age; speak and read English; be a non-smoker; be free of taste deficits; oral disorders; oral piercings; not be pregnant or nursing (for female subjects). The study was approved by the Oregon State University Internal Review Board. Four different sessions were conducted in March, April and May of 2015. Each individual participated in two 30 min sessions. Triangle tests were separated into 3 sets, each set containing the 6 triangle tests using the same model wines. A total of 50% of the participants had their first session with 2 sets and second session with 1 set and the other 50% had 1 set in their first session and 2 sets in their second sensory session. Sets were served in random order. Triangle tests were presented starting with the low concentration of β-ionone or β-damascenone followed by increasing concentrations to reduce/prevent fatigue. Half of the participating individuals started with β-ionone wines first and half of the individuals started with β-damascenone wines. Individuals had one-minute breaks between each triangle test and a three minute break after six triangle tests to avoid fatigue. Panelists were presented with a paper ballot and were asked to circle the number that corresponded with the sample they thought smelled different. They smelled samples from left to right but were allowed to go back and smell samples within the triangle test as needed.

### 4.6. Descriptive Analysis

The 15 samples of treatments that were determined as being significant from the consumer panels (Table 1 and Table 2) were presented to 7 professional winemakers (2 women, 5 men) from Oregon for descriptive analysis. The β-ionone screening samples were first presented to the winemakers, to determine their sensitivity to β-ionone. All seven could detect β-ionone at both low and high concentrations. A total of 16 samples (15 wine samples and a warm up wine) were analyzed by the winemakers, presented in random order. A glass of water and unsalted saltine crackers were provided for the winemakers to cleanse their palette as needed. A one minute break took place between each wine sample and a 5 min break after the 6th and 12th sample to avoid fatigue. The panelists were asked to mark on 100 mm line scales to evaluate the intensity of 5 aromas; red berry, dark berry, floral, vanilla, and other. These descriptors were chosen based off of previous research [16,22]. The “other” category was provided so as to track changes in aroma composition that may not be inherent to the typical Pinot noir aroma and allow for the winemakers to thoroughly describe the aroma of each sample. All results were recorded on paper ballots.

### 4.7. Chemical Analysis

Samples were analyzed by ETS laboratories (St. Helena, CA, USA) using their Rapid phenolic panel and general wine chemistry panel.

### 4.8. Statistical Analysis

Statistical significance for triangle tests were determined using signal detection theory [29]. Two way ANOVA was used for the descriptive analysis of data to determine the effects and interaction of wine and compound concentration on sensory perception using XLStat Sensory (version 2020.3.1, Addinsoft, New York, NY, USA).

## 5. Conclusions

The objective of this study was to determine the potential effect of β-ionone and β-damascenone on Pinot noir wine aroma quality. It was found that assessors could detect low concentrations of β-ionone in all three model wines. The effect of β-damascenone was not clearly indicated, as perception of the compounds at the various concentrations varied across the three model wines. Paired with the descriptive analysis results, it is clear that there is a strong matrix effect for the perception of these two compounds, which not only alters the concentration at which they are perceived, but also the way in which the aroma is described. It was found that the anecdotal suggestion that higher concentrations of β-ionone and β-damascenone—above the levels normally found in wine—negatively affect wine, is misguided, and that the concentration at which these compounds start to negatively impact the aroma of wine has yet to be determined. Further research should take place in order to determine the matrix interactions that alter the perception of wine aromas, as this will be necessary to be able to predict aromas from chemical data.

## Figures and Tables

**Table 1:** *p*-values for β-ionone concentration calculated with signal detection theory.

Model Wine	β-Ionone (μg/L)	d-Prime	*p*-Value
1 †			
	0.1	1.66	<0.0001
	1.5	2.46	<0.0001
	6	2.19	<0.0001
2 ‡			
	0.1	0.95	0.0351
	1.5	1.26	0.0024
	6	1.79	<0.0001
3§			
	0.1	1.4	0.0007
	1.5	1.68	<0.0001
	6	2.37	<0.0001

† Oregon wine #1. ‡ Oregon wine #2. § New Zealand wine.

**Table 2:** Significance of β-damascenone concentration on Pinot noir wine aroma using signal detection theory.

Model Wine	β-Damascenone (μg/L)	d-Prime	*p*-Value
1 †			
	2	1.4	<0.0006
	6	1.11	<0.0099
	21	1.66	<0.0001
2 ‡			
	2	0.76	NS *
	6	>0.3333	NS
	21	1.4	0.0006
3 §			
	2	0.72	NS
	6	1.68	<0.0001
	21	2.09	<0.0001

† Oregon wine #1. ‡ Oregon wine #2. § New Zealand wine. * NS = non significant.

**Table 3:** *p*-values for ANOVA Type 1 sum of squares analysis for each aroma attribute.

Source of Variance	Vanilla	Floral	Dark Berries	Red Berries
Compound † and concentration ‡	0.274	0.070	0.004 **	0.394
Model Wine	0.046 *	0.245	0.222	0.356
Compound and concentration * model wine	0.388	0.258	0.021 *	0.271

* α = 0.05, ** α = 0.01, † = β-ionone or β-damascenone, ‡ = low, high or 3×.

**Table 4:** Basic wine chemical and phenolic analysis of the model wines.

Analysis	MW1	MW2	MW3
Chemical (mg/L)			
Free sulfur dioxide	7	5	3
Molecular sulfur dioxide	<0.10	<0.10	0.10
Total sulfur dioxide	45	33	23
Titratable acidity (g/L)	5.8	5.8	6.4
pH	3.86	3.55	3.51
Volatile acidity (acetic, g/L)	1.03	0.90	0.63
Phenolics (mg/L)			
Catechin	124	65	83
Total tannin (epicatechin equiv.)	269	385	295
Polymeric Anthocyanins	7	13	12
Total anthocyanins	206	82	46
Catechin/tannin index	0.461	0.169	0.281
Polymeric anthocyanins/tannin index	0.026	0.034	0.041

## Sample Availability

Samples of the compounds are not available from the authors.
